# Detection of oncogenic virus genomes and gene products in lung carcinoma

**DOI:** 10.1038/sj.bjc.6602409

**Published:** 2005-02-08

**Authors:** L Brouchet, S Valmary, M Dahan, A Didier, F Galateau-Salle, P Brousset, B Degano

**Affiliations:** 1 Department of Respiratory Diseases, CHU Rangueil-Larrey, Toulouse, France; 2 Department of Pathology, Purpan Hospital and INSERM U563 (CPTP), CHU Purpan, Toulouse, France; 3 Department of Pathology, CHU Caen, France

**Keywords:** lung cancer, oncogenic viruses, immunohistochemistry, *in situ* hybridisation, tissue microarrays

## Abstract

We investigated a series of 122 cases of small cell lung carcinomas and non-small cell lung carcinomas for the presence of several viruses that are known to be oncogenic in humans. Thus, viral genomes (DNA) and/or RNA transcripts and/or proteins of human papillomaviruses (HPV) 16, 18, 31, 33, 51, Epstein–Barr virus (EBV), human herpesvirus 8 (HHV-8), human cytomegalovirus (HCMV) and simian virus 40 (SV40) were investigated on tissue sections (prepared in tissue microarrays) with different techniques of immunohistochemistry and *in situ* hybridisation. None of the cases displayed a single positive tumour cell for all the viruses tested whatever the technique applied. Of note, in five cases of tumours with lymphoid infiltrates, we detected scattered EBV (EBER)-positive bystander lymphocytes. In three cases, a faint nuclear staining was found with the anti-latent nuclear antigen/LANA1 (HHV-8) antibody. These cases were checked by PCR with two sets of primers (orf 26 and orf 75) and remained negative for this latter virus. Taken together, our data strongly suggest that the conventional human oncogenic viruses (HPV, EBV, HCMV, HHV-8 and SV40) are unlikely to play some role in the development of lung carcinomas.

A few virus species have been detected in human cancers. In some human tumours, these viruses probably play a critical role in carcinogenesis since they are present early during the process of cancer development and are constantly detectable in the tumour cells. Among these, human T-cell leukaemia virus-1 (HTLV-1) (Matsuoka, 2003), Epstein–Barr virus (EBV) (Macsween and Crawford, 2003), human herpesvirus-8 (HHV-8) (Ganem, 1997) and human papillomaviruses (HPV) (zur Hausen, 2000) are now well recognised as oncogenic and are specifically associated with different types of tumours. Other viruses have been incriminated in human carcinogenesis but there is still a hard debate regarding their direct implication in cancer, in particular concerning simian virus 40 (SV40) (Carbone *et al*, 2003) and human cytomegalovirus (HCMV) (Cobbs *et al*, 2002).

Large body of data have demonstrated the presence of SV40 in different tumours in humans, but the majority of the studies have relied on PCR detection of the viral genome (Bergsagel *et al*, 1992; Galateau-Salle *et al*, 1998; Martini *et al*, 1996). There are still controversies as to whether SV40 is directly linked to cancer development. Early studies have shown that SV40 genome was present in mesothelioma (Carbone *et al*, 1997), but immunodetection of SV40 as a diagnostic marker of human mesothelioma is not carried out in routine pathology (Carbone *et al*, 2003).

Recent results have implicated HCMV in the pathogenesis of malignant gliomas (Cobbs *et al*, 2002), prostate cancers (Samanta *et al*, 2003) and colon cancers (Harkins *et al*, 2002; Samanta *et al*, 2003). The human cytomegalovirus is a *β*-herpesvirus able to infect various types of human cells, including glial cells, endothelial cells and epithelial cells (Hummel and Abecassis, 2002). As other herpesviruses, HCMV infects 80% of the adult human population and persists in reservoir cells throughout life. Nevertheless, this virus is not yet recognised as an oncogenic factor, in particular *in vivo* (Hsu *et al*, 2004).

The epidemiology of lung cancers remains partially unresolved since the vast majority of tobacco users do not develop such tumours while at least 10–15% of lung cancers occur in non smokers (Alberg and Samet, 2003). Thus, factors other than smoking may also have an impact as aetiological and risk factors for lung cancer. Different studies have suggested that the HPVs (Syrjanen, 2002), EBV (Grinstein *et al*, 2002) and to a less extent SV40 (Galateau-Salle *et al*, 1998) may be involved in lung cancer development, but the results are discrepant or underline some geographical variations. A viral aetiology of lung tumours is tantalising since the virus can be regarded as a main oncogenic event (like HPV in genital tract) or as an important cofactor as in human mesotheliomas (SV40 plus asbestosis).

In this study, we tried to address the question of a viral aetiology of lung cancers by investigating a series of 122 cases of small cell lung carcinomas (SCLC) and non-small cell lung carcinomas (NSCLC) with immunohistochemistry and *in situ* hybridisation for the presence of five different virus species (HPV, EBV, HHV-8, SV40, HCMV).

## MATERIALS AND METHODS

### Tissue samples

Two tissue microarrays (TMAs) were used for this study. The array blocks were constructed using a technique previously described (Charafe-Jauffret *et al*, 2003) with a tissue arrayer from Beecher Instruments (Beecher Instruments, Micro-array technology, Sun Prairie, WI, USA). Cores (0.6 mm) were taken from paraffin-embedded SCLC and NSCLC retrieved from our files at the Purpan Hospital in Toulouse between 1999 and 2002. Each tumour was represented by two cores. A total of 40 tumour samples were classified as squamous cell carcinoma (SCC), 31 as adenocarcinoma (AC), five as AC-SCC, four as large-cell lung cancer, nine as small-cell lung cancer, 13 as large-cell neuroendocrine carcinoma, seven as bronchioloalveolar carcinoma, three as atypical bronchial carcinoid, six as typical bronchial carcinoid and four as pleomorphic carcinoma.

The approval of the French equivalent of the Institutional Review Board (Comité Consultatif de Préservation des Personnes en Recherche Biologique/CCPPRB) was not required for such an investigation based on archived paraffin blocks that have been routinely used for diagnostic purposes.

### Immunohistochemistry

#### Antibodies

*Anti-CMV*: We used an anti-HCMV monoclonal antibody (clone E13, Argene-Biosoft, Varihes, France). It recognises an immediate early antigen of HCMV (IE1) and does not cross react with EBV, adenovirus, varicella-zoster virus and HSV. It works very well in positive controls (working dilution 1 : 100) and gives a strong nuclear staining in tissues (colitis) with active HCMV infection. In addition, a weak cytoplasmic staining is seen in virtually all infected cells. The staining of the positive controls was obtained after standard antigen retrieval and/or after amplification of the signal by catalysed system amplification (CSA, Dako, Carpintera, CA, USA).

*Anti-SV40*: An anti-Tag antibody from Oncogene Research Products (San Diego, CA, USA) was used. This antibody (Ab-2, Clone Pab416) worked well on paraffin sections of tissues fixed either in 10% buffered formalin or in Bouin's liquid. The staining of the positive control cell line was obtained after standard antigen retrieval and/or after amplification of the signal by CSA. One hepatocellular carcinoma cell line from T-Ag transgenic mice (Weber-Benarous *et al*, 1993) and a case of renal infection (Li *et al*, 2002) were used as positive controls. For negative controls (DNA negative), five tissue sections from T-Ag PCR-negative lung tumours and non-neoplastic tissues were used.

*Anti-HHV-8*: The rat monoclonal anti-LANA-1 antibody (LN53) (ABI, Columbia, MD, USA) was used at a dilution of 1 : 2000 according to a previously published protocol (Brousset *et al*, 2001). This antibody stains latently infected HHV-8-positive cells (Kaposi's sarcoma) and the signal is nuclear with a stippling pattern.

*Anti-EBV*: Two monoclonal antibodies (anti-EBNA2, clone PE2, and anti-LMP1, clone CS1-4, Dako, Carpinteria, CA, USA) were used as described in detail elsewhere (Brousset *et al*, 1994). Lymphatic tissue from a patient with infectious mononucleosis was used as positive control.

*Anti-HPV*: We used the anti-VP1 capsid protein (clone K1H8) monoclonal antibody (Dako, Carpinteria, CA, USA). This antibody recognises a nonconformational internal linear epitope of the major capsid protein VP1. K1H8 antibody works well on paraffin section and in thin layers of cervical smears, and detects various types of viral strains (in particular, HPV type 6, 11, 16, 18, 31, 33, 42, 51, 52, 56 and 58) (Rouyer *et al*, 2004).

#### Immunohistochemical procedure

Immunostaining on paraffin sections was performed using the method described elsewhere with little modifications (Brousset *et al*, 2001). Briefly, paraffin sections were mounted on glass slides coated with silane (Sigma Chemical Co, Saint Quentin, France). Sections were deparaffinized, placed in 10 mmol L^−1^ Na-citrate buffer (pH 6) and heated in a microwave oven (Whirlpool model; Philips, Eindhoven, Holland) at 900 W for cycles of 20 min and 10 min. The slides were removed from the oven and allowed to cool for 30 min at room temperature. After washing in water, endogenous peroxidase was blocked with 1% hydrogen peroxide in methanol for 30 min. Slides were then rinsed in PBS before staining with a streptavidin–biotin three-stage technique, with the DAKO Strept ABC complex/HRP Duet kit (Dako, code no. K492, Carpinteria, CA, USA). In parallel, amplification with catalysed system amplification (CSA, Dako, Carpinteria, CA, USA) was applied according to the supplier's recommendations. The Dako CSA system HRP is an extremely sensitive immunohistochemical staining procedure incorporating a signal amplification method based on the peroxidase catalyzed deposition of a biotinylated compound followed by a secondary reaction with streptavidin peroxidase (Dako, Carpinteria, CA, USA).

### *In situ* hybridisation

*Human cytomegalovirus*: This technique has been described elsewhere (Brousset *et al*, 1992). A biotinylated DNA probe from a commercial source (Enzo, PathoGene DNA probe assay, CMV DNA probe pack, part no. 32802, Enzo Life Sciences Inc., Faimingdale, NY, USA) was used according to the manufacter's recommendations with some modifications. In our hands, there was a clearcut correlation between *in situ* hybridisation and immunohistochemistry for control tissues.

*Epstein–Barr virus*: We used the peptidic nucleic acid (PNA) EBER1 probes from Dako (Carpinteria, CA, USA) according to the manufacturer's recommendations.

*Human papillomavirus*: Probes against strains 16-18-31-33-51 (from Enzo Life Sciences Inc., Faimingdale, NY, USA) were used and applied with the same procedure as for HCMV.

#### PCR

For PCR detection of HHV-8, amplification was performed with standard protocols with DNA extracted from formalin-fixed paraffin-embedded tissues. We chose the primer sets within orf 26 and orf 75 as previously described (Brousset *et al*, 2001). The other viruses were not tested since they are ubiquitous and detectable as bystanders in virtually all tissues tested.

## RESULTS

### Immunohistochemistry

All the antibodies against the specific proteins of the five viruses tested in this study gave negative results in lung tumour tissues. All controls tested in the same conditions stained reproducibly. Two lymph nodes with infectious mononucleosis contained EBNA2 (PE2)- and LMP1 (CS1-4)-positive immunoblasts. Two lung biopsies from patients with active HCMV replication contained numerous IE1 (E13)-positive cells. Two cases of Kaposi's sarcoma and one case of multicentric Castleman's disease were repeatedly positive with anti-LNA1 antibody. Three cervical biopsies with condyloma were used as positive controls for anti-HPV (K1H8) antibody. Lastly, the two controls used to validate the anti-SV40 Tag antibody were positive in each experiment without using the amplification (CSA) technique.

None of the cases of lung cancer included in this study contained a single positive tumour cell with all the above-referenced antibodies. In three cases of SCC, we found a faint nuclear staining with the anti-LANA1 (HHV-8) antibody. This signal was weak and diffuse and lacked the characteristic stippling pattern. Nevertheless, the absence of viral infection was confirmed by PCR with two sets of primers (orf 26 and orf 75) (not shown).

### *In situ* hybridisation

The results were very close to those of the immunohistochemistry in that no tumour cell was detected with any of the probes. Of note, in five cases with important lymphoid infiltrates (three AC, two SCC), a few scattered small bystander lymphocytes were detected with the EBER1 probes. We failed to detect a single positive case with the several anti-HPV probes we used.

## DISCUSSION

Besides some cases associated with HPV and rare cases infected by EBV, the incidence of viral infection in lung carcinomas remains marginal. We developed different techniques of detection of viruses already incriminated in lung tumours (HPV and EBV) and we extended our survey to viruses more recently implicated in cancer (SV40, HCMV and HHV-8). We have been unable to detect a single case (out of 122) positive for any of the five different viruses tested.

To be suspected to play some role in human tumours, an oncogenic virus should be specifically detected in the tumour cells. This theory also implies that the virus is present in all cells of a tumour in a monoclonal fashion, suggesting that the viral infection is an early event consistent with a clonal expansion of the tumour cells (Brousset *et al*, 1994) . In addition, a few viruses like EBV, HCMV and sometimes HPV are ubiquitous and infect a high percentage of people, rendering PCR investigations of the tumours poorly reliable as PCR products can be yielded from bystander infected cells. This is frequently the case with EBV (Meggetto *et al*, 1997) and also with HPV (Pett *et al*, 2004) and possibly SV40.

The detection rates of HPV in lung carcinomas are subject to wide variations. In a recent review of 85 studies recording about 2500 cases, Syrjanen (2002) described a detection rate varying from 0 to 100%. We failed to reproduce results obtained by other groups who used the same techniques as ours, that is, immunohistochemistry and *in situ* hybridisation. This discrepancy may be explained at least in part by geographical epidemiological variations since most studies with positive results for HPV were performed in Asia (Miyagi *et al*, 2000; Cheng *et al*, 2001). However, our results are also discrepant with those of the Finnish group of Syrjanen *et al* (1989), who found a detection rate of 7% for HPV among 131 patients with lung cancers. In order to clarify this difference, it may be useful to look for the presence of HPV in lung cancers of nonsmoking patients, where HPV could be found more frequently as cocarcinogen. In our study, only four out of 122 patients were nonsmokers.

Epstein–Barr virus probably plays a sporadic role in lung cancers (Grinstein *et al*, 2002). Only a few cases of EBV-associated tumours have been reported in the literature. We failed to detect EBV antigens in epithelial cells of our samples. EBV antigens were investigated with the anti-EBNA2 and anti-LMP1 antibodies because of a good sensibility and reproducibility of staining in control slides with an adapted technique of antigen retrieval. Moreover, the negativity of *in situ* hybridisation with EBER is the strongest argument for the absence of the latently infected cells in our tumours. Although our series is possibly biased since we had only two cases with lymphoepithelial architecture, we can deduce from our data that EBV is not at play in the most frequent types of lung cancers.

Simian virus 40 genome and gene products have been detected by some groups in mesotheliomas (Carbone *et al*, 2003). From an epidemiological point of view, variation in positivity between studies may be related to great geographical variation of SV40 contamination of people through poliomyelitis vaccination. Indeed, it is not possible to identify precisely individuals who were exposed to SV40, as few vaccine batches were tested for contamination, not all batches were contaminated and distribution of contaminated batches from one country to another was not controlled (Gazdar *et al*, 2002). Some authors have suggested that the viral genes, in particular Tag, were weakly expressed in mesotheliomas (Butel and Lednicky, 1999). However, immunodetection of SV40 Tag is not commonly recognised as a diagnostic marker enabling the distinction between mesothelioma and adenocarcinoma, or between mesothelioma and reactive mesothelial hyperplasia (Jasani, 1999). The lack of reproducibility of immunohistochemistry is difficult to explain since there are specific anti-Tag monoclonal antibodies that work well on paraffin sections making it possible the detection of the virus in physiological conditions. All of our cases have been tested in parallel with a highly sensitive amplification method of the immunohistochemical signal (CSA method), and a signal was obtained with the positive controls only. Galateau-Salle *et al* (1998) have reported the presence of SV40 DNA sequences in mesotheliomas and also in lung cancers and in benign (inflammatory) conditions as well. By immunohistochemistry, we have investigated four cases of SV40-positive lung tumours from this latter study and we obtained negative results in all instances (data not shown). Last but not least, Lopez-Rios *et al* recently published against a role for SV40 infection in human mesothelioma. In more than 70 mesothelioma samples, there lacked T-Ag-positive staining, such as in our experiments. The authors also performed DNA extraction and PCR, and found positive results only when primers were in the region of T-Ag gene (nucleotides 4100–4713) that is present in laboratory plasmids. Conversely, PCR were negative when primers were not included within that region (Lopez-Rios *et al*, 2004).

The negativity we found for HHV-8 is expected since so far this virus has not been associated with epithelial tumours. Human cytomegalovirus is not a good candidate in lung carcinogenesis as well. Indeed, one group only has reported on the detection of this virus in CNS tumours (Cobbs *et al*, 2002) and also in colonic (Harkins *et al*, 2002) and prostate cancers (Samanta *et al*, 2003). As far as the CNS tumours are concerned, we have been unable to reproduce the results of the study by Cobbs *et al*. We have found that the virus could be detected in only a few cases of CNS tumours not in malignant cells but rather in putative stromal cells (Sabatier *et al*, 2005; data not shown).

The major conclusion of our study is that the main oncogenic viruses usually found in solid tumours in humans are not implicated in the vast majority of lung carcinomas.
